# Nonlinear SAR Modelling of Mosquito Repellents for Skin Application

**DOI:** 10.3390/toxics11100837

**Published:** 2023-10-02

**Authors:** James Devillers, Adeline Larghi, Valérie Sartor, Marie-Laure Setier-Rio, Christophe Lagneau, Hugo Devillers

**Affiliations:** 1 CTIS, 69140 Rillieux-La-Pape, France; 2 EID Méditerranée, Direction Technique, 34184 Montpellier, France; 3 Laboratoire des IMRCP, Université de Toulouse, CNRS UMR 5623, Université Toulouse III-Paul Sabatier, 31062 Toulouse, France; 4 SPO, University Montpellier, INRAE, Institut Agro, 34000 Montpellier, France

**Keywords:** repellent, mosquitoes, *Aedes aegypti*, Structure–Activity Relationship (SAR), artificial neural network

## Abstract

Finding new marketable mosquito repellents is a complex and time-consuming process that can be optimized via modelling. In this context, a SAR (Structure–Activity Relationship) model was designed from a set of 2171 molecules whose actual repellent activity against *Aedes aegypti* was available. Information-rich descriptors were used as input neurons of a three-layer perceptron (TLP) to compute the models. The most interesting classification model was a 20/6/2 TLP showing 94% and 89% accuracy on the training set and test set, respectively. A total of 57 other artificial neural network models based on the same architecture were also computed. This allowed us to consider all chemicals both as training and test set members in order to better interpret the results obtained with the selected model. Most of the wrong predictions were explainable. The 20/6/2 TLP model was then used for predicting the potential repellent activity of new molecules. Among them, two were successfully evaluated in vivo.

## 1. Introduction

Mosquitoes (Diptera: Culicidae) can be the vectors of severe human pathogens such as the etiologic agents of malaria, dengue, Zika, and yellow fever, leading to high rates of morbidity and mortality worldwide [1]. As a result, for example, more than 3.9 billion people in over 129 countries are at risk of being contaminated by the dengue virus, with an estimated 96 million symptomatic cases and about 40,000 deaths every year [2].

In any case, the transmission cycle requires that a vector meets a contaminated host. This occurs via a blood meal, during which the mosquito can acquire viruses or protozoa together with blood from an infected host. Pathogens reproduce within the mosquito digestive tract, and after a defined time migrate to the salivary glands. At this stage, the mosquito becomes able to transmit the pathogen to a susceptible host in a subsequent bite [2,3]. Blood-feeding behaviour, density, and longevity are key factors that govern the ability of mosquito populations to spread pathogens [4]. As a result, repellents are used as a vector control strategy to avoid mosquito bites and thereby disrupt the chain of the transmission of pathogens [5,6]. More generally, repellents also allow for a reduction in the nuisance caused by mosquitoes at high population densities. 

The number of mosquito repellents commercialized for vector control is limited; among them, DEET (*N*,*N*-diethyl-*m*-toluamide or *N*,*N*-diethyl-3-methylbenzamide, CAS RN: 134-62-3) is considered the gold standard of repellents, because it is highly effective against most mosquito species and provides longer protection time [7]. However, it presents some shortcomings, such as an unpleasant odour and damaging reactions with certain rubber and plastic materials [7,8]. Consequently, there is an urgent need to find new mosquito repellents that provide long-lasting action, have limited toxicity, and are eco-friendly.

The discovery process of new mosquito repellents can be hastened by using the (Quantitative) Structure–Activity Relationship ((Q)SAR) methodology [9,10]. It is based on the idea that the activity of an organic molecule depends on its structure or parts thereof. Thus, briefly, a set of molecules with their activity values obtained under the same experimental conditions are described by molecular descriptors encoding physicochemical properties or/and topological indices and/or structural features. A linear or nonlinear statistical approach is then used to relate the activity values to the selected descriptors. After a validation process, the obtained model can be used for predicting the activity of untested molecules only from their descriptor values [9]. The QSAR methodology is commonly used in the discovery of new drugs and for estimating the toxicity of chemicals (see, e.g., [11,12,13,14]). It has been also applied to the identification of new mosquito repellents to apply on the skin and fabrics [15,16,17]. Whatever the endpoint of concern, the main limiting factors in QSAR modelling are very often the quality of the activity data and the size of the training set [9]. The latter factor was clearly identified by Devillers [15] in the critical analysis of published SAR and QSAR models aiming to predict skin repellent compounds. The poor structural diversity of the training samples was also stressed as a factor limiting the capacity of the models to propose new structures with good potential repellent activity [15].

In this context, the goal of our study was to propose a Structure–Activity Relationship (SAR) model, allowing for the prediction of repellents for skin application. A large structurally diverse dataset of 2171 chemicals with their repellent activity were used. These molecules were described with molecular descriptors encoding physicochemical properties, topological information, and structural features. A powerful nonlinear supervised machine learning method was used for computing classification models with acceptable prediction performances. Finally, the selected model was used for proposing candidate molecules whose repellent activity was validated in vivo for some of them.

## 2. Materials and Methods

### 2.1. Mosquito Repellent Activity

The results of tests performed on *Aedes aegypti* under the same experimental conditions were retrieved from Knippling et al. [18]. Briefly, chemicals were tested by evenly distributing 1 mL over the forearms of two to four men. Gloves were worn to protect the hands and the arms were exposed in test cages containing 2000 to 4000 unfed mosquitoes of various ages [18]. Treated arms were exposed in the cages for three minutes at intervals of about 30 min until the first bite was received. It is noteworthy that the mosquitoes were discarded when the biting rate on an untreated arm in a half-minute was below 20, whereas it was usually from 50 to 75. The tests were performed in rooms maintained at a temperature of 27–29 °C and a relative humidity of 80–85% [18].

The time between the treatment and the first bite was used as a criterion of effectiveness and was referred to as the protection time. Chemicals that repelled for less than 60 min, 61 to 120 min, 121 to 180 min, and more than 180 min were allocated to the classes 1, 2, 3, and 4, respectively. To facilitate the search for relationships between the structure of the chemicals and their repellent activity, classes 2 and 3 were not considered, and only classes 1 and 4 were selected for the design of SAR models. Inorganic chemicals, oils, and mixtures were discarded. In the same way, chemicals cited twice, with an ambiguity in their chemical name, or with missing information (e.g., the location of a substituent) were also eliminated. This led to a total of 2171 chemicals. Among them, 318 were allocated to class 4 and 1853 to class 1. Table S1 (Supplementary material) lists the 2171 chemicals with their chemical name and experimental activity value [18]. Subsequently, classes 1 and 4 were conveniently termed classes 0 and 1, respectively. The dataset of 2171 molecules was randomly split into training and external test sets of 80% and 20%, respectively.

### 2.2. Molecular Descriptors

RDKit software (http://www.rdkit.org/, accessed on 6 June 2023), version 2021-03-4, was used for computing 53 descriptors selected for their clear interpretability. They encoded physicochemical properties, topological information (e.g., size, branching), and the presence of specific atoms, functional groups, and structural features. No selected descriptor showed an occurrence of less than 5% in the whole dataset. Although the RDKit software also allowed for the calculation of the 1-octanol/water partition coefficient (log P) of the organic molecules, the MlogP model [19,20] included in VEGA 1.1.4 (https://www.vegahub.eu/portfolio-item/vega-qsar/, accessed on 6 June 2023) was selected because it was successfully used in our previous study [16]. In addition, the training and test sets of the MlogP model are available, and there are parameters allowing us to estimate the reliability of the prediction results.

### 2.3. Nonlinear Supervised Machine Learning Method

A three-layer perceptron (TLP) [21] was used for modelling the skin repellent activity of chemicals against *Ae. aegypti*. The input layer consisted of the selected molecular descriptors. The hidden layer included an adjustable number of neurons that needed to be reduced as much as possible to avoid overfitting problems. The output layer comprised two neurons corresponding to the activities (0 and 1).

The hyperparameter setting was as follows: The sum of the squares and the cross entropy were tested as the error function. The activation functions on the hidden and output neurons were either identity, logistic, tanh, or exponential. It is noteworthy that with the cross-entropy error function, the output activation function was always set to softmax. This restriction ensures that the TLP outputs are true class membership probabilities; this is known to increase the performance of the classification neural networks. All the calculations were performed with the data mining module of the Statistica™ software version 12 (StatSoft, Inc., Tusla, OK, USA). The TLP of this software package includes two biases. Although different algorithms were available for training the learning set of a TLP, the Broyden–Fletcher–Goldfarb–Shanno (BFGS) algorithm was selected because this second-order optimization algorithm guarantees fast convergence. Lastly, it is important to note that the molecular descriptors (input neurons) were scaled using linear transformation such that the original minimum and maximum of every descriptor was mapped to the range (0,1).

### 2.4. Performance Evaluation Metrics

The classification performances of the computed TLP models were assessed from the calculation of the sensitivity or true positive rate, the specificity or true negative rate, and the accuracy. The F1 score, the Matthews correlation coefficient (MCC), G-mean, and dominance were also calculated. Additionally, the receiver operating characteristic (ROC) curve and the area under the ROC curve (AUC) were computed from the PRROC package version 1.3.1 (https://cran.r-project.org/, accessed on 6 June 2023). The full definitions of the parameters above and the criteria for their interpretation have been thoroughly described in Devillers et al. [16].

### 2.5. In Vivo Evaluation of Repellent Activity

Some compounds that were predicted as potential repellents by the selected TLP model were evaluated for their in vivo repellent activity by using the tube test system of Grieco et al. [22] taken up by the World Health Organization (WHO) [23]. The purpose of this test was to assess the repellent potential of chemicals to the *Ae. aegypti* Bora Bora strain in comparison with DEET used as a reference compound. 

Briefly, Whatman^®^ Chromatography n°1 papers (15 × 12 cm) were uniformly impregnated with a solution at a given percentage (or mg/m^2^) of the chemical of interest dissolved in acetone. An in-house software (graciously provided by IRD, Montpellier, France) accounting for the molecular weight of the studied chemical and its purity percentage was used for preparing the solution at the desired concentration and for a specified number of papers (i.e., 4). The papers were dried for 5 min before being packaged in a plastic sheet. They were used the same day.

The sensitive Bora Bora strain has been reared for many years at the EID Méditerranée insectarium under strictly controlled conditions (27 °C (±2), 70% (±10) relative humidity, 16/8 L/D photoperiod). A total of 25 unfed 5–11-day old female mosquitoes were collected using a mouth aspirator and inserted into one tube of the WHO apparatus. A control tube containing a blank paper (acetone only) was screwed to the tube containing the mosquito females. A second tube containing the paper impregnated with the compound solution to be tested was added at the other end of the tube containing the mosquitoes. Thus, the tube containing the batch of mosquito females was in a central position, and it was gated at its two extremities with drawers. The test device was set up on a stable support and left for 30 s without intervention to allow the mosquitoes to acclimatize. The tubes were arranged randomly, with the treated side facing the operator in order to avoid any attractive effect. The drawers were then opened simultaneously and after 10 min, closed simultaneously. Once neutralized by the cold (freezer), the number of mosquitoes per compartment was then counted. Three replicates (3 × 4) were carried out at different days for each tested chemical. 

The actual repellency potential of the candidate molecules was then estimated by calculating the spatial activity index (SAI) [22] (Equation (1)).

SAI = (Nc − Nt) / (Nc + Nt) (1)

In Equation (1), Nc is the number of mosquitoes in the control chamber, and Nt is the number of mosquitoes in the treated chamber. 

WHO [23] recommends a second index (Equation (2)) taking into account the mosquitoes remaining in the central tube. This index was also calculated.

SAI_W_ = ((Nc − Nt) / (Nc + Nt)) × (Nm /N) (2)

In Equation (2), Nm is equal to Nc + Nt, and N is the total number of mosquitoes in the test apparatus.

The spatial activity index varies from −1 to 1. Zero indicates an absence of response, whereas –1 indicates that all the mosquitoes moved into the treatment chamber, resulting in an attractant response; 1 reveals that all the mosquitoes moved into the control chamber, resulting in a spatial repellent response.

## 3. Results and Discussion

The selection of the most informative descriptors for classifying the molecules experimentally identified as repellents and non-repellents in the laboratory tests was made from the Feature Selection option of the data mining module of the Statistica™ software version 12 (StatSoft, Inc., Tusla, OK, USA) and by considering the modelling results obtained in our recent study [16]. 

Selecting the best TLP architecture as possible requires a compromise to be found between complying with the necessary principle of parsimony on the one hand [24] and having enough input neurons (i.e., descriptors) to correctly describe the structural and functional characteristics of the molecules, as well as an optimal number of hidden neurons to distribute the information within the network on the other hand. The task is even more delicate when, as is the case here, the dataset is not balanced, with 318 repellents and 1853 molecules that are not repellents. 

From the numerous trials made by randomly selecting different training and test sets of 80%/20%, we showed that the input neurons needed to include physicochemical descriptors and topological indices [25], as well as descriptors encoding structural features. Thus, TLP models including 17 to 22 input neurons and 5 to 8 hidden neurons were experienced. The cross entropy with tanh as the hidden activation function and softmax as the output activation function often led to much better results than the sum of squares with identity, logistic, tanh, or exponential as activation functions. Thus, among the different runs analysed, the most interesting model was a 20/6/2 TLP with the cross entropy as the error function and tanh and softmax as hidden and output activation functions, respectively. The convergence of the BFGS algorithm was obtained in 95 cycles. The input neurons included three physicochemical descriptors (PDs), seven topological indices (TIs) [25], and ten constitutional descriptors (CDs). The PDs were the molecular weight (MolWt), the polar surface area of the molecule (TPSA), and the 1-octanol/water partition coefficient (MlogP) calculated according to Moriguchi et al. [19,20]. The TIs were the Balaban J index (BalabanJ) [26], able to account for small structural changes between similar structures; the Bertz index (BertzCT) [27], which quantifies the complexity of molecules; the molecular connectivity indices of the first order (Chi1n) and the second order of valence (Chi2v) [28], which encode mostly the size but also the degree of the skeletal branching of the molecules; and the α value (HallKierAlpha) and the Kappa indices of order 1 (Kappa1) and 2 (Kappa2) [29], which describe the shape and flexibility of the molecules. The ten CDs were the number of saturated rings (NumSaturatedRings), the number of aliphatic hydroxyl groups (fr_Al_OH), the number of aliphatic hydroxyl groups excluding the *tert*-OH (fr_Al_OH_noTert), the number of carbonyl O (fr_C_O), the number of tertiary amines (fr_NH0), the number of amides (fr_amide), the number of esters (fr_ester), the number of ether oxygens—including phenoxy (fr_ether), the number of ketones (fr_ketone), and lastly, the number of methoxy groups (fr_methoxy). It is worth noting that among the selected molecular descriptors, 16 were already selected in our previous model [16].

**Figure 1:**
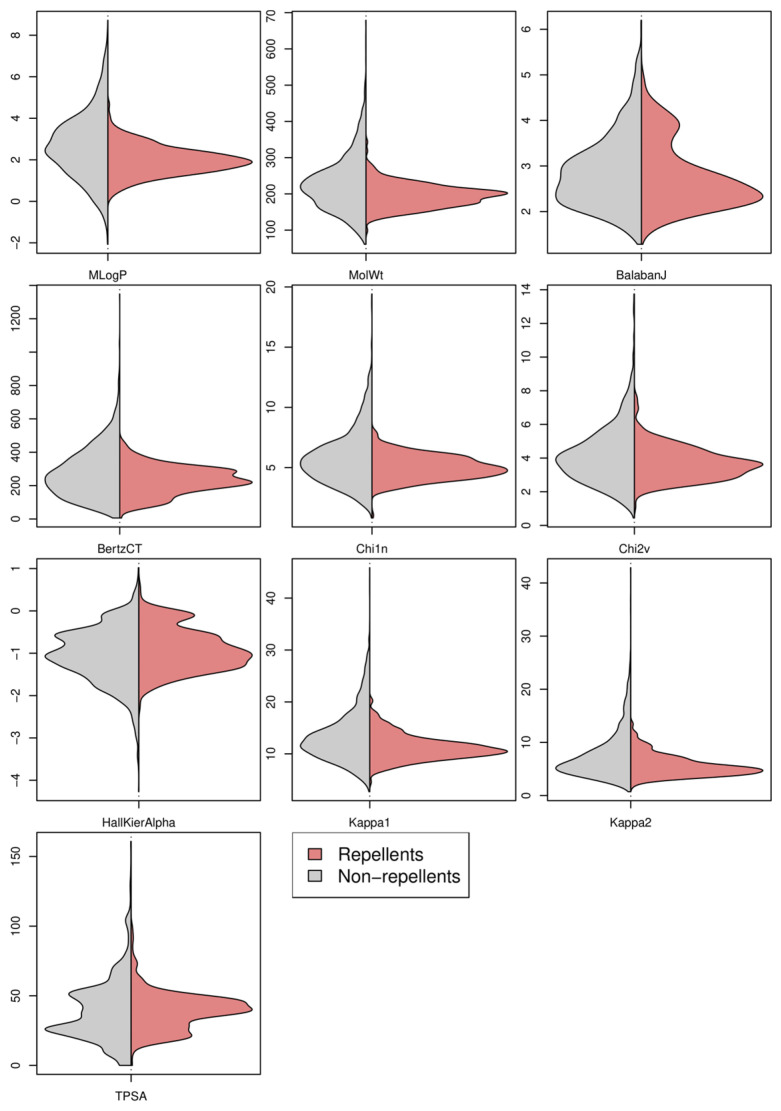
Density distribution of the 10 continuous descriptors used as input neurons in the selected TLP model. The non-repellents (**left**) and repellents (**right**) are represented in grey and red, respectively.

**Figure 2:**
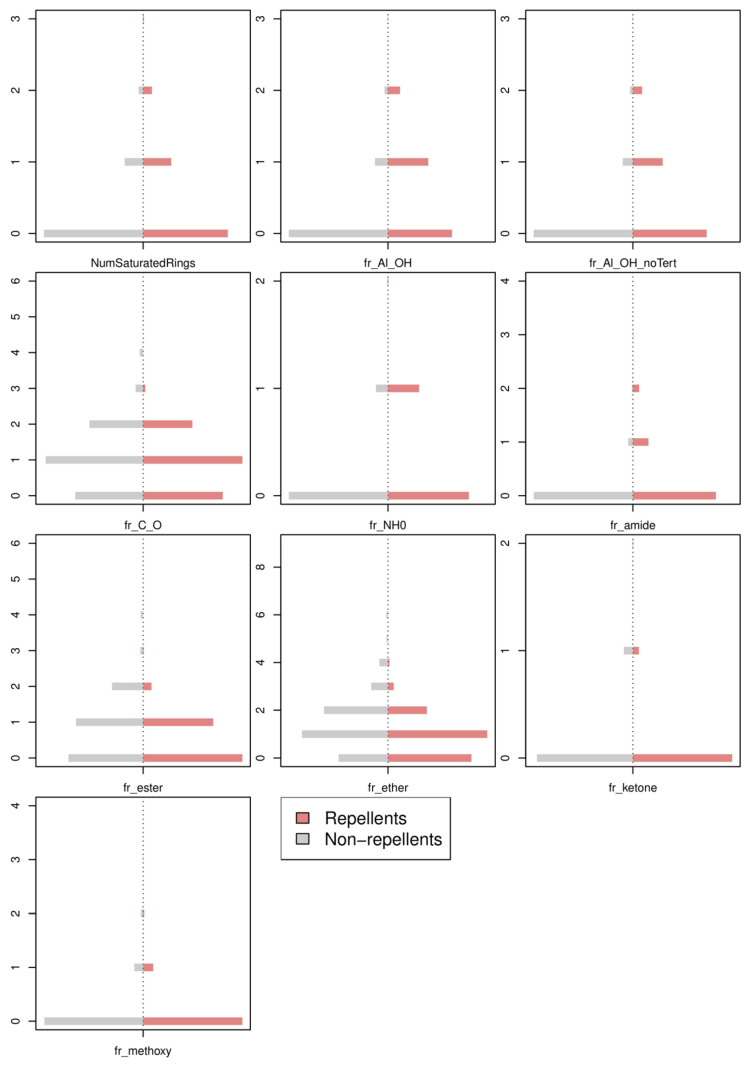
The distribution of the 10 discrete descriptors used as input neurons in the selected TLP model. The non-repellents (**left**) and repellents (**right**) are represented in grey and red, respectively.

In Figure 1 and Figure 2, the range of values of each descriptor used as input neurons of the selected TLP model was represented for the whole dataset by distinguishing between the molecules that were experimentally measured as repellents and those as non-repellents. The analysis of the 20 corresponding maps is information rich. Figure 1 shows the density distribution of the 10 continuous variables (PDs and TIs). For each descriptor, the areas under the density curves are identical between the repellents and the non-repellents for facilitating the comparison of the distribution of their values. An inspection of Figure 1 shows that the spanning of the descriptor values for the non-repellent molecules is often much larger than for the repellents. It is especially true for some descriptors. Thus, the MlogP values for the repellents are distributed over a narrow range compared with those of the non-repellent compounds. This finding has already been highlighted in the literature. Indeed, Suryanarayana et al. [30] showed that among a series of amides, a better repellent activity was obtained in molecules having log P values ranging between 1.5 and 2.5. The same trend is observed with MolWt (Figure 1), and it was also stressed by Rayner and Wright [31]. From 50 structurally diverse molecules that were identified as highly effective repellents in both the skin and in cloth tests, these authors showed that their molecular weights (MWs) ranged with only one exception from 146 to 257. At the opposite, the MWs of 80 compounds detected as non-repellents with both tests showed values ranged from 71 to 679 with one indeterminate (a polymer) [31]. On the other hand, in Figure 1, it is interesting to underline that for some descriptors, their ranges of values are broadly the same, irrespective of the activity of the molecules. 

The Balaban J index (BalabanJ) fully illustrates this situation. Although this topological index is very interesting for describing and discriminating molecules due to its low degeneracy [26], it needs to be coupled with other descriptors to lead to QSAR and QSPR (Quantitative Structure–Property Relationship) models with acceptable predictive performances [32,33,34,35].

Figure 2 shows the distribution of the 10 discrete variables (CDs) used as the input neurons of the selected TLP model. Counts were scaled by the total number of molecules of each category (i.e., 1853 non-repellents and 318 repellents) to make the pairs of distributions comparable. An inspection of Figure 2 reveals that the absence of a descriptor in a molecule could make it either a repellent or a non-repellent. Conversely, when a selected descriptor is found once or twice in a molecule, this favours repellent activity. The descriptors fr_Al_OH, fr_Al_OH_noTert, fr_NH0, and fr_amide illustrate this trend. When the number of occurrences of a descriptor is superior to two, it is generally detrimental to repellent activity. This tendency is exemplified in Figure 2 with the descriptors fr_C_O, fr_ester, and fr_ether. Although these 10 discrete descriptors are very interesting for encoding the molecules studied, Figure 2 clearly shows that they could not be used alone for classifying the molecules into repellents and non-repellents, because each of them only accounts for a structural part of a molecule. These descriptors are information rich as regards the understanding of the mechanisms of action of the molecules, but the price to pay is the need to consider TLP models with a rather high number of input neurons.

The confusion matrices obtained with the selected TLP model for the training, test, and whole sets of molecules are shown in Table 1, and the performance metrics calculated from the three sets are given in Table 2. An inspection of these tables shows that 94% of the molecules belonging to the training set have their activity correctly predicted by the selected 20/6/2 TLP model. The results are a little bit worse as regards the test set.

**Figure 3:**
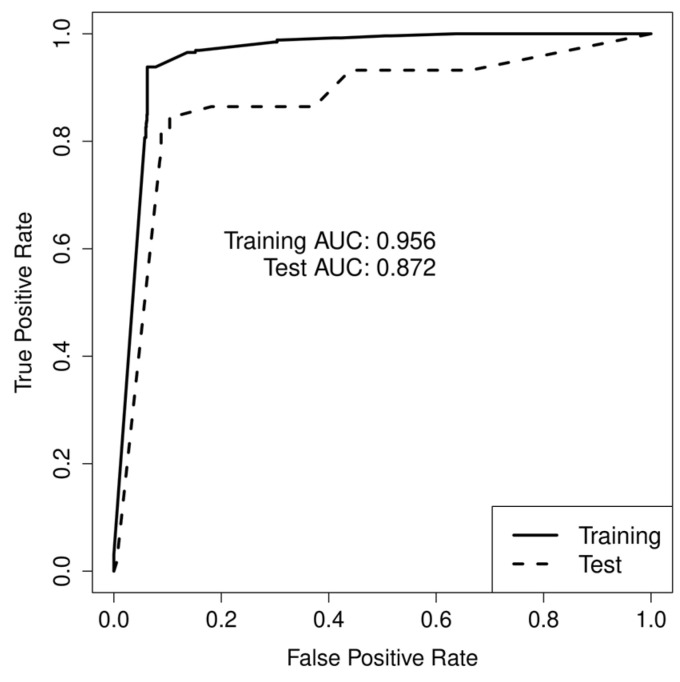
Receiver operating characteristic (ROC) curve and area under the ROC curve (AUC) for the training and test sets of the selected TLP model.

Indeed, Table 2 shows that 89% of the test set compounds are correctly predicted by the model. However, a specificity of 90% is obtained with the test set. It is also interesting to note that the dominance values for the training set and test set are equal to 0 and −0.05, respectively (Table 2). The dominance parameter takes values ranging from −1 to +1. A value of +1 reveals a perfect accuracy on the positive class but failing on all negative cases while −1 corresponds to the converse. The closer the value is to 0, the more balanced both individual rates are [36,37]. The receiver operating characteristic (ROC) curve and area under the ROC curve (AUC) for both sets confirm this difference in the prediction performances between the training and test sets (Figure 3). Nevertheless, an analysis of the other evaluation parameters calculated for both sets (Table 1 and Table 2)—and even more importantly, the predicted activities calculated with the selected TLP model (Table S1)—confirm the acceptable prediction performances of the model.

When selecting a model, it is crucial to determine the structural scaffolds and functional groups in the molecules that are very often correctly predicted from those which, on the contrary, are most often poorly predicted. To do so, it is necessary to statistically analyse the results obtained with different training and test sets. Thus, the dataset of 2171 molecules was randomly split 57 times into training and external test sets of 80% and 20%, respectively. The 57 different training sets were used to compute 20/6/2 TLPs by using the input neurons, learning algorithm, and activation functions of the selected model. The percentages of good predictions obtained for both sets are given in Table S1 along with the number of times each chemical of the whole dataset was randomly allocated to the test set. These 57 splits allowed us to consider all the chemicals at least once as training set and test set members. Table S1 shows that this number ranges from 2 (#1259 (*o*-*n*-hexyloxybenzaldehyde) and #1267 (alpha-hydroxy-beta-acetoxy-isobutyric acid, *iso*-amyl ester)) to 25 (#2067, thiocyanic acid, benzhydryl ester). An inspection of Table S1 shows that 1301 compounds (59.93%) are always correctly predicted by the 57 TLPs whether belonging to the training sets or to the test sets. With regard to the non-repellents, 1166 are always correctly predicted as such from a total of 1853 in the whole dataset (62.92%). Regarding the repellents, 135 are always correctly predicted from a total of 318 in the whole dataset (42.45%). Obviously, the percentages of good predictions increase when the threshold values are lower than 100%. Thus, for example, if we consider the configurations in which at least 90% of good predictions are obtained for the training set compounds and at least 85% for the test set compounds, there are 1654 chemicals (76.19%) that satisfy both criteria simultaneously. Keeping at least 90% of good predictions for the training set compounds but less than 85% for the test set compounds, the number of compounds drops to 173 (i.e., 7.97%). However, an inspection of Table S1 also shows that the repellent activity of some chemicals is very often or always incorrectly predicted by the TLPs. Thus, *N*-acetyl-1,2,3,4-tetrahydroisoquinoline (#152), *p*-*iso*-butoxybenzyl alcohol (#385), *N*-butyl-*N*-(2-acetoxyethyl)acetamide (#404), *N*,*N*-diethyl-alpha-toluamide (#918), 2,6-dimethyl-1,2,3,6-tetrahydrobenzaldehyde allyl acetal (#990), *N*,*N*-dipropylsuccinamic acid, methyl ester (#1039), *o*-ethoxyphenethyl alcohol (#1080), and *p*-*iso*-propoxybenzyl alcohol (#1929) have their activity always incorrectly predicted whether belonging to the training or test sets. The repellency potential of these chemicals is also wrongly predicted by the selected model. These eight molecules are non-repellents predicted as repellents by the selected model and the other 57 models because there are close structures in the dataset that are repellents and are predicted as such by the models. Thus, for example, *N*-acetyl-1,2,3,4-tetrahydroisoquinoline (#152) is a non-repellent always predicted as being a repellent because *N*-acetyl-1,2,3,4-tetrahydroquinoline (#151) is a repellent always predicted as such by the models (Table S1). It is interesting to note that *N*-acetyl-1,2,3,4-tetrahydroquinoline was also found to be a repellent on fabrics [18] and was predicted as such by all our previous models computed from physicochemical descriptors, topological indices, and structural features [16]. This was also very often the case when fingerprints were used for encoding the structure of the molecules [17]. *N*-acetyl-1,2,3,4-tetrahydroisoquinoline (#152) did not belong to the previous dataset [16,17]. 

In the dataset, there are 150 molecules, including at least a chlorine atom in their structure, and among them, only nine (6%) were experimentally identified as repellents (#623, #644, #671, #673, #677, #1115, #1737, #2166, and #2169). The 141 non-repellent chlorine molecules of the dataset were always correctly allocated to class 0 by the selected TLP model, except compounds #625, #661, #670, and #1490. 2-(2-Chloro-4-ethyl-phenoxy)ethanol (#661) belongs to the test set of the selected model. As such, it was always incorrectly classified by the 57 models. When it belongs to the training sets, only 11.9% of good predictions are obtained. The situation is better as regards chloroacetic acid, 2-phenoxyethyl ester (#625) and beta-methyl-beta-*p*-chlorophenylglycidic acid, ethyl ester (#1490)—which belong to the test set of the selected model—as well as *o*-chlorophenoxyacetic acid, ethyl ester (#670), which is a training set compound (Table S1). Regarding the chlorinated molecules identified as repellents, chloroacetic acid, 2-methyl-2-nitropropyl ester (#623), 2-(4-chlorophenoxy)ethanol (#671,) and 2-(4-chlorophenyl)ethanol (#673) were always correctly allocated to class 1 by the selected model and the other 57 models whether belonging to the training or test sets (Table S1). Phenylacetic acid, beta-chloroallyl ester (#1737) was correctly allocated to class 1 by the selected TLP model, but with the 57 other models, only 88.24% and 66.67% of good classifications are obtained for the training and test sets, respectively. *p*-Chlorobenzylfurylcarbinol (#644) and ethylene dichloride (#1115) are training set compounds wrongly classified by the selected model. An analysis of the predictions obtained with the 57 models shows that the former chemical is correctly allocated to class 1 in 82.98% of cases when it belongs to the training sets and 60% when it is included in the test sets. The latter molecule was correctly predicted only in 62.75% and 16.67% of the cases when it belongs to the training and test sets, respectively (Table S1). The repellents #677 (beta-chloropropionic acid, tetrahydrofurfuryl ester), #2166 (*o*-chloro-alpha-(trichloromethyl)benzyl alcohol), and #2169 (9-hendecenoic acid, 1-trichloro-2-methyl-2-propyl ester) are always identified as non-repellents when they belong to the test sets of the 57 models. As training set members, they were correctly identified at 12.5%, 67.92%, and 95.83% regarding #2169, #677, and #2166, respectively. These three compounds were also wrongly predicted by the selected model, even if chemicals #2166 and #2169 belong to its training set (Table S1). 

Most of the time, the model is able to take into account the changes in activity linked to the variation in the number and the position of carbon atoms of an aliphatic chain of an ester. Thus, for example, anthranilic acid, methyl ester (#242), ethyl ester (#240), *n*-propyl ester (#243), and *iso*-propyl ester (#244) are repellents identified as such by the selected model. In the same way, anthranilic acid, *iso*-amyl ester (#238), cyclohexyl ester (#239), and *n*-hexyl ester (#241) are not repellents and were correctly allocated to class 0 by the selected model. Very often, these compounds are also correctly classified by the 57 models (Table S1). On the contrary, there are cases where the model is unable to take into account the structural particularities present on a given molecular scaffold. Thus, for example, *N*,*N*-diethylsuccinamic acid, ethyl ester (#914), *n*-propyl ester (#916), *iso*-propyl ester (#917), and *sec*-butyl ester (#913) are repellents identified as such by the selected model and the 57 models whether belonging to the training sets or test sets (Table S1). *N*,*N*-diethylsuccinamic acid, 2-methoxyethyl ester (#915) is not a repellent. It was correctly allocated to class 0 by the selected model, for which it acted as a training set member. An analysis of the 57 models shows that 52.38% and 33.33% of good predictions are obtained when the chemical is included in the training and test sets, respectively. *N*,*N*-diethylsuccinamic acid, allyl ester (#910), *n*-butyl ester (#911), and *iso*-butyl ester (#912) are not repellents. These chemicals are never correctly identified when they belong to the test sets. At best, about 2% of good predictions are obtained when they are included in the training sets and consequently, it is not surprising that the selected model was not able to allocate them to class 0, even if they were all training set members.

Nevertheless, these results confirm that the selected descriptors and TLP architecture are suited for modelling the dataset.

**Figure 4:**
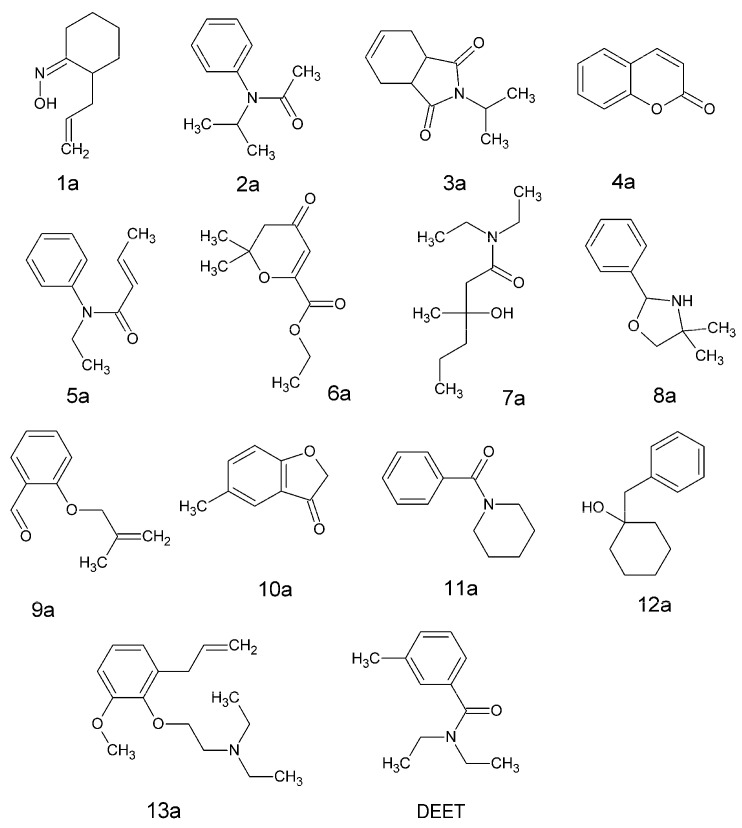
The structures of compounds #**1a** to #**13a** and DEET.

Knippling et al. [18] tested the repellent activity of both 4137 chemicals after their application to the skin and 3239 chemicals after fabric impregnation. We found interesting to focus on the chemicals not evaluated with the skin test but found to have the highest repellent score (i.e., class 4) after their application to clothes to show how the selected TLP model predicted their repellent activity on skin. Some illustrative examples are given in Figure 4. Thus, 2-allylcyclohexanone oxime (compound #**1a** in Figure 4) was allocated to class 1 by the selected model. It is interesting to note that 4-methyl cyclohexanone oxime (compound #1506 in Table S1) is a repellent that belongs to the test set of the selected model, and it was incorrectly classified by the model while it was very often correctly predicted by the 57 other models whether belonging to the training or test sets. To avoid confusion, it is important to remember, as indicated in the Section 2.1 that an allocation to class 1 with the selected model corresponds to a class 4 in Knippling et al. [18].

*N*-*iso*-propylacetanilide (compound #**2a**) was identified as a repellent by the selected model. Interestingly *N*-*n*-amylacetanilide (#200) is a repellent correctly predicted by the selected model and at 97.96% by the 57 models when it belongs to the training sets. It was always correctly predicted as a test set member. In the same way, *N*-*iso*-amylacetanilide (#201) is a repellent always predicted as such by all the models (Table S1). These two molecules were also experimentally identified as repellents for clothing application [18]. It is noteworthy that Paluch et al. [38] recognized *N*-butylacetanilide as a mosquito repellent. *N*-*iso*-propyl-1,2,3,6-tetrahydrophthalimide (compound #**3a** in Figure 4) was allocated to class 1 by the selected TLP model. This prediction must be compared to those obtained for the *N*-*n*-propyl- (#1966), *N*-butyl- (#463), *N*-*iso*-butyl- (#464), and *N*-*sec*-butyl-(#465) derivatives of 1,2,3,6-tetrahydrophthalimide, which are repellents and were always allocated to class 1 by all the models whether belonging to the training or test sets (Table S1). However, notice that although *N*-amyl-1,2,3,6-tetrahydrophthalimide (#231) is not a repellent, it was allocated to class 1 by the selected model and very often by the 57 other models (Table S1). *N*-*n*-propyl-4-methyl-1,2,3,6-tetrahydrophthalimide, and *N*-*iso*-propyl-4-methyl-1,2,3,6-tetrahydrophthalimide were also allocated to class 1 by the selected model (structures not displayed).

Lastly, an inspection of Table S1 reveals that *N*-amyl-4-methyl-1,2,3,6-tetrahydrophthalimide (#218), *N*-beta-butyroxyethyl-1,2,3,6-tetrahydrophthalimide (#529), and *N*-beta-hydroxypropyl-4-methyl-1,2,3,6-tetrahydrophthalimide (#1321) are not repellents and they were identified as such by the selected model. While compound #529 was very often allocated to class 0 by the other 57 models, it was not the case as regards compounds #218 and #1321 (Table S1). Thus, effects on the repellency of a methyl substitution on the 1,2,3,6-tetrahydrophthalimide scaffold are influenced by the number of carbons of the N alkyl chain. The presence of hetero atoms or functional groups on the N alkyl chain seems to be detrimental to repellent activity. 

Coumarin (compound #**4a** in Figure 4) was identified as a repellent by the selected model. This is in agreement with the results found by Tunon et al. [39] on this mosquito species. A biting-deterrent effect against *Ae. aegypti* was also noted by Cantrell et al. [40]. Lastly, it is interesting to note that coumarin was also found to be a repellent against the nymphs of the tick *Ixodes ricinus* [39].

*N*-ethylcrotonanilide (compound #**5a** in Figure 4) was identified as a repellent by the selected TLP model. This prediction result has to be compared in the light of those obtained for the molecules of the dataset having the same structural scaffold. *N*-methylcrotonanilide (#1499) is a repellent that is included in the test set of the selected model. It was always predicted as such by all the models. This chemical was also found to belong to the highest class of repellency following the fabric impregnation test [18]. *N*-*n*-butylcrotonanilide (#418) is a non-repellent compound that was correctly predicted by the selected model as training set member. However, it was not always allocated to class 0 with the 57 other models, especially when categorized as a test set member. Indeed, in that case, the success of classification was only 45% (Table S1). Knippling et al. [18] did not evaluate the potential repellent activity of this molecule on clothes.

Mesityl oxide oxalic acid, ethyl ester (compound #**6a** in Figure 4), also termed ethyl indalone, was allocated to class 1 by the selected model. It is interesting to note that mesityl oxide oxalic acid, *n*-butyl ester (also named indalone) was experimentally tested by Knippling et al. [18] on both the skin and on cloth. A result corresponding to class 3 was obtained with the former test and to class 4 with the latter. Interestingly, the selected TLP model identified indalone as a repellent. Indalone is one of the oldest patented and used mosquito repellents [41]. Robert et al. [42] showed that indalone was a better repellent than DEET against *Anopheles albimanus*, but the 95% confidence interval found was high, revealing some variability in the results [42]. Fossati and Maroli [43] also found that the repellent activity of indalone was more effective than that of DEET against *Phlebotomus perniciosus*.

*N*,*N*-diethyl-3-hydroxy-3-methyl caproamide (compound #**7a**), 4,4-dimethyl-2-phenyl-tetrahydrooxazole (compound #**8a**), *o*-methallyloxybenzaldehyde (#**9a**), 5-methyl-3(2)-benzofuranone (#**10a**), *N*-benzoylpiperidine (#**11a**), and 1-benzylcyclohexanol-1 (#**12a**) in Figure 4 were all identified as good repellents when applied to clothes (class 4) and were also classified as good skin repellents by the selected TLP model.

As proof of concept, the repellent activity of compound #**12a** was measured in vivo by using the test system of Grieco et al. [22]. The chemical was purchased from Sigma-Aldrich (95% purity) and used without further purification. For comparison purposes, DEET (Abcr, 97% purity) was also tested. It is noteworthy that DEET (Figure 4) was identified as a repellent (class 1) by the selected TLP model. The experimental results obtained for both compounds are shown in Table 3. Although their SAI values are equivalent, the SAIw value of chemical #**12a** outperforms the one of DEET, revealing slightly better repellent activity against *Ae. aegypti* for that compound.

**Figure A1:**
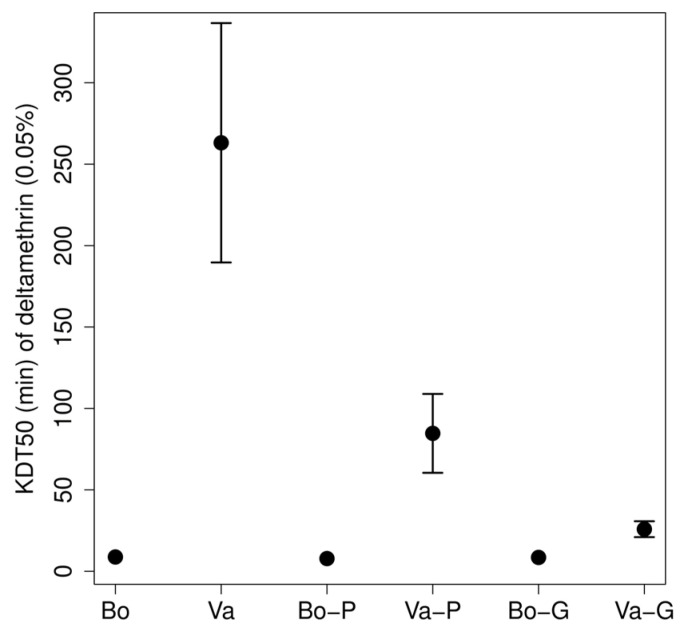
KDT50 (min) of deltamethrin (0.05%) on the sensitive Bora Bora strain (Bo) and resistant Vauclin strain (Va) of *Ae. aegypti* and after the addition of PBO (P) at 0.087 mg/cm^2^ or gravitol (G) at 0.067 mg/cm^2^.

Recently, a QSAR model was built to find new synergists, increasing the efficacy of deltamethrin against pyrethroid-resistant *Ae. aegypti* mosquitoes [44]. Among the predicted potential candidates, the most interesting was the so-called PSM-05 molecule that is known under the tradename of gravitol (CAS RN: 6006-09-3). Indeed, Devillers et al. [44] showed that gravitol was more effective than piperonyl butoxide (PBO) in increasing the toxicity of deltamethrin against the Vauclin strain of *Ae. aegypti* that is highly resistant to pyrethroids [45,46,47]. The key results are summarized in Appendix A (Figure A1). Because gravitol (compound #**13a** in Figure 4) shows the structural attributes of aromatic repellent molecules, it was interesting to investigate whether gravitol was a true repellent or not. Interestingly, the selected TLP model allocated the molecule to class 1. Thus, the actual repellent activity of gravitol was fully tested in vivo against the sensitive Bora Bora strain of *Ae. aegypti*. The results obtained are listed in Table 3. Based on the SAI and SAI_w_ values and those of their corresponding confidence intervals, it appears that molecule #**13a** exhibits a repellent activity equivalent to that of DEET. 

Although gravitol shows interesting properties against *Ae. Aegypti*, its potential toxicity against non-target insects should also be evaluated. Due to the ecological importance of bees and their sensitivity to pollutants [48], the oral and contact toxicity of gravitol to the honeybee was estimated with BeeToxAI [49]. The result was negative for both routes of exposure. The nontoxicity of gravitol against the honeybee was also confirmed by BeeTox [50], another QSAR model based on another methodology than BeeToxAI.

Even if there is no ambiguity that the in silico repellent activity of molecules #**12a** and #**13a** against *Ae. aegypti* was confirmed in vivo, the results obtained must be considered with care due to the margin of uncertainty related to the type of laboratory test used. Indeed, due to financial constraints, it was unfortunately not possible to use arm-in-cage tests [51] to evaluate the repellent activity of the candidate molecules. In addition, the demonstration of a repellent activity against *Ae. aegypti* does not guarantee that the other mosquito species of medical interest will also behave similarly.

Today, mosquito resistance to insecticides jeopardizes all the efforts made in vector control. Consequently, there is a need to find new insecticides with different mechanisms of action that are better equipped to deal with the problems of mosquito resistance to insecticides [52]. In this context, it would be interesting to more thoroughly evaluate the synergy potential and repellent activity of gravitol as well as its ecotoxicity.

Lastly, it is noteworthy that the selected TLP model was used to find original chemical structures with a potential repellent activity. Some of them are currently synthetized to be further tested in the laboratory.

## 4. Conclusions

The use of skin repellents against mosquitoes is an efficient self-protection practice for the prevention of bites. This may also lower the rate of vector disease transmission. To date, the number of marketed products that repel mosquitoes is low, and there is room for new and effective repellents showing safer profiles that could circumvent the problem of efficacy decrease [53,54]. 

Modelling approaches are suited to reach this goal. They allow us to discover original structural scaffolds with a repellent activity and also better understand the mechanisms of action of new and existing repellents [15,55,56]. 

In this study, a neural network classification model was built from a dataset of 2171 molecules, for which their actual repellent activity was measured under the same experimental conditions on *Aedes aegypti* female mosquitoes. The molecules were described by means of physicochemical and topological descriptors and by descriptors encoding the presence of specific atoms, functional groups, and structural features. Eighty percent of the dataset (i.e., 1737 molecules) were randomly selected for computing the models. The remaining molecules were used for estimating the performances of the models. Classical statistical metrics were also employed for selecting the best model that was a 20/6/2 TLP. The twenty input neurons included three physicochemical descriptors, seven topological indices, and ten constitutional descriptors in relation to the structure of the molecules. The dataset was also randomly split 57 times into training and external test sets (80% vs. 20%) to compute 57 20/6/2 TLPs by using the input neurons, learning algorithm, and activation functions of the selected model. As such, the molecules were distributed to the external test sets in a range of 2 to 25 times. This allowed for a better interpretation of the performances of the selected model, which were acceptable, since 94% and 89% of the good predictions were obtained on the training set and test set, respectively. The selected TLP model was used for predicting the repellent activity of 13 additional molecules and DEET. In vivo confirmation was performed on *Ae. aegypti* for two of them and for DEET. Among them, gravitol deserves further investigation, as the molecule was also shown to be a good synergist for increasing the efficacy of deltamethrin against pyrethroid-resistant *Ae. aegypti* mosquitoes.

Our future work will consist of more thoroughly exploring the simulation predictions of the selected model in order to propose more structurally diverse candidates and validate in vivo their repellent activity. In addition, while the 1-octanol/water partition coefficient (log P) can be related to the repellent activity of groups of molecules [30,57], it is well known that the predicted log P value of a molecule can significantly vary according to the log P model used [20,58,59]. Consequently, the influences of such changes on the prediction results will also be investigated.

## Figures and Tables

**Table 1:** Confusion matrices obtained with the selected TLP model for the different sets of molecules.

Dataset	TP	TN	FP	FN	TTP	TTN
Training set	243	1386	92	16	259	1478
Test set	50	336	39	9	59	375
Whole set	293	1722	131	25	318	1853

TP = True positive, TN = True negative, FP = False positive, FN = False negative, TTP = Total positive, TTN = Total negative.

**Table 2:** Performance metrics calculated from the selected TLP model for the three sets of molecules.

Metrics	Training Set	Test Set	Whole Set
Sensitivity	0.94	0.85	0.92
Specificity	0.94	0.90	0.93
Accuracy	0.94	0.89	0.93
F1	0.82	0.68	0.79
MCC	0.79	0.63	0.76
AUC	0.96	0.87	0.94
G-mean	0.94	0.87	0.93
Dominance	0	−0.05	−0.01

**Table 3:** The prediction of the repellent activity of DEET and molecules **12a** and **13a** against *Ae. aegypti*.

Molecule	SAI *	Conf. int.	SAI_W_	Conf. int.
DEET (7%)	0.46	0.28–0.64	0.28	0.16–0.39
12a (7%)	0.47	0.32–0.61	0.38	0.25–0.51
13a (7%)	0.45	0.32–0.59	0.32	0.21–0.43

* SAI and SAI_w_ values were calculated according to Equation (1) and Equation (2), respectively (see text).

## Data Availability

Data used to generate the models are available in Knippling, E.F.; McAlister, L.C.; Jones, H.A. Results of Screening Tests with Materials Evaluated as Insecticides, Miticides, and Repellents at the Orlando, Fla., Laboratory, April 1942 to April 1947, USDA Publication E-733, United States Department of Agriculture, Agriculture Research Administration, Bureau of Entomology and Plant Quarantine, Orlando, FL, USA, 1947. The other data can be found in the manuscript or the Supplementary Material.

## Supplementary Materials

The following supporting information can be downloaded at: https://www.mdpi.com/article/10.3390/toxics11100837/s1, Table S1: The list of molecules with their observed (ACT) and predicted (Pred.) activities (0 = not repellent; 1 = repellent) obtained with the selected TLP model and the percentages of good predictions obtained with the different training and test sets of the 57 models.
